# Hospital and Institutional News

**Published:** 1920-09-25

**Authors:** 


### HOSPITAL AND INSTITUTIONAL NEWS.

### New Policy of the Birmingham Hospital League.

THE General Hospital League at Birmingham was originally formed to relieve the grave financial position of the General Hospital, now faced by an accumulated deficiency of £65,000. It has now been decided, we are happy to announce, to widen the scope of the movement so as to embrace any of the other hospitals of the city which desire to be included in the movement. The *Birmingham Mail* very sensibly says:

It is no secret that the decision will remove very grave objections which were entertained in many quarters to the original proposal, and which had been voiced within the League itself and by a very emphatic resolution of the Hospital Council. No one wanted to stand in the way of a special effort and appeal on behalf of the General Hospital, but it was felt that a scheme like this, so far-reaching in its scope, and aiming particularly at securing regular and permanent contributions from the workers and others, would be likely to damage the prospects of the other hospitals whenever they might find it necessary to go to the public with a similar appeal.

The scheme depends for its success upon the industrious and intensive culture of the whole field open for raising a harvest of hospital subscriptions. It is the intention of its promoters, by utilising every available means of propaganda, to bring the urgent needs of the voluntary hospitals before everybody, and to enlist sustained support. At present there are scores of thousands of people, by no means confined to the working classes, who give little or nothing to these institutions, which draw their funds mainly from a limited list of wealthy and generous citizens and from a large, but by no means complete, body of the workers. If the Hospitals League can spread its net widely enough to cover all, can induce everyone to contribute something according to his or her means, whether it be a penny or a shilling a week, or a more or less substantial annual subscription, all the money needed will be forthcoming.

### A HUMANE SCIENCE.

THE possibility that the near future will see the foundation of a definite school of industrial medicine, which would be an integral part of London University (St. Mary's Hospital), is one which all should view with the greatest satisfaction, and one for which, if opportunity occurs, national assistance is more than deserved. The investigations carried on during the war upon subjects somewhat vaguely classed as industrial fatigue have already resulted in a number of general hygienic improvements in the conditions of industrial life, but in so short a time it cannot be expected that more than a tithe of the necessary work has been accomplished. We have here again a type of research which, requiring as it does the readily available assistance of all the ancillary sciences, must be centralised in its study and must be established in close connection with some great seat of scientific and economic learning. If practical and valuable results are to be obtained financial support must be forthcoming. Though a comparatively new science, its future goes hand in hand with a new and better conception of industrial relationships as a whole, and should bring inestimable advantages, not only to the worker, but to the nation in its broadest sense.

### VENTILATION AND OPEN AIR.

WE would call particular attention to the recently published Part II. of "The Science of Ventilation and Open-air Treatment," by Professor Leonard Hill, Director of the Department of Applied Physiology under the Medical Research Council. Obtainable from H.M. Stationery Office through the usual channels, this volume forms a magnificent complement to Part I. of the same work, and though again, in places, almost alarmingly technical, its pages should be consulted by all who take pleasure in carrying out health work by methods other than those of rule-of-thumb. Sections are devoted to some of the most fascinating subjects of the times, and their study makes one realise only too well how superficial has been our general teaching concerning them. Some of these topics may at first sight appear almost hackneyed, but by the time the end of a chapter is reached the reader will hold far other opinions. "The Penetration and Action of Light on Living Organisms," "The Colour of the Skin and Racial Adaption to Climate," "Fevers and their Open-air Treatment," "Concerning Clothes," "Food, Exercise, and Climate" are all, to mention but a few of them, dealt with in a truly masterly fashion. In coming issues of this journal we shall attempt to summarise several of the most outstanding investigations, but to those who have the time and a true scientific interest in their surroundings we would strongly recommend a first-hand perusal of No. 52 of the Council's special report series.

### CENTRALISATION IN LONDON.

THE September number of the *Hospital Saturday Fund Journal* contains an article by Mr. Harold Spender advocating the "reform" of the London hospitals by the establishment of a Central London Hospitals Board. As a former member of the Hospitals Committee of the Metropolitan Asylums Board, he is prepossessed in favour of a centralised control for the distribution of patients, the placing of contracts, and the control of the medical services. The plan has always had its advocates, and also its difficulties. Such attempts as have been made in its direction have hitherto not had much success. But apart from them co-ordination has been growing, and if existing lines are followed the fruits of co-ordination may be much increased, without the sacrifice of those separate characters of which most of the great hospitals are jealous. The line of least resistance is to encourage the tendency to co-operation and to see how far it can be carried. We believe that any cut-and-dried scheme for a Central Board would frighten institutions away from it in the same way that amalgamation creates countless critics in most of the larger centres where it has been advocated.

### THE BOLINGBROKE HOSPITAL.

We are happy to learn from Mr. A. J. Oakley, Secretary-Superintendent of the Bolingbroke Hospital, that the bank overdraft has been reduced within six months from £9,000 to £4,000. It was in May last only that we referred to the heavy debt that hampered the Committee in its efforts, and expressed the hope that the workpeople of the neighbourhood would take a larger view of their responsibilities and come forward to help their local hospital. We are glad to learn that the action of the local employees in increasing their weekly contribution from 1d. to 2d. has in a large measure helped to bring about the result that we refer to. We congratulate the hospital on its improved outlook.

### THE NEW SECRETARY AT BIRMINGHAM.

Mr. Hedley Lucas, superintendent and secretary of the Royal Albert Edward Infirmary, Wigan, and formerly assistant secretary and superintendent of the King Edward the Seventh Hospital, Cardiff, has been appointed superintendent and secretary of the Queen's Hospital, Birmingham. Mr. Lucas has been at Wigan nearly three years, during which time the institution's income has risen from £12,000 to £22,000.

### AN AMERICAN AND AN ENGLISH HOSPITAL COMPARED.

The beautifully printed and illustrated annual report of the Massachusetts General Hospital opens with a paragraph which is at once humorous and a sign of the times. The trustees point out that even the most needed of charities must economise in these times of the reduced buying power of the dollar. Every effort is made to fit the expenditure to the income, without allowing the patients to suffer. "This necessitates a most delicate adjustment; for who can define the meeting-point of pure science and applied science?" We are greatly struck by the very large cost of maintaining a hospital in Boston as compared with one in London, England. The Massachusetts General Hospital has 332 beds. The salaries and wages account comes to just under £91,000 (including administration salaries, £13,500). The food bill was £46,400 in 1919. For the same year at St. Thomas's Hospital, with 632 beds, a sum of £52,222 was paid for salaries and wages and £39,949 for food. We have taken the old rough exchange value of five dollars to the pound, but, whichever way one looks at it, the comparison is remarkable.

### ANOTHER FLAG DAY.

Civic authorities are uniting forces on behalf of the Imperial War Relief Fund, in order to assist the League of Nations to stem the typhus menace, and the effort will take the form of a flag day in London on Saturday, October 2, of which the organising secretary is Mr. G. A. G. Paterson, Fishmongers' Hall. The number of typhus cases in Poland and Galicia alone has increased from 34,538 in 1916 to 231,206 in 1919, and it is estimated that the total will be about 360,000 this year. Military events in Poland have made matters worse, first by disarranging certain preventive measures already taken, and second by exposing the source of infection in Russia. The funds now requested are for clothing, hospital equipment, foodstuffs, soap and bathing plant, disinfecting apparatus, motor transport, and medical personnel. Members of the League of Nations have been asked by the Council to contribute £2,000,000 for the relief of typhus in Poland.

### THE BUCKS HOSPITAL SECRETARYSHIP.

Mr. Stanley E. Wilkins has resigned the secretaryship of the Royal Bucks Hospital, Aylesbury, which he has held for fourteen years. Mr. Percy Cutting, secretary to the Bucks War Pensions Committee, is succeeding him as honorary secretary.

### A MINERS' HOSPITAL AT CAERPHILLY.

A hospital provided by miners exclusively for miner patients will shortly be opened. When "The Beeches," the residence of a mining contractor, standing in its own grounds about a quarter of a mile from Caerphilly Railway Station, became available recently it was purchased for £5,400 by the East Glamorgan District of Miners. A proposal that it be converted into a modern hospital, at a cost, including furniture, of £30,000, has now received the support of all the miners in the district, who have agreed to contribute sixpence per week towards carrying out the alterations. The hospital is to comprise twenty-one beds, and is to be known locally as the Caerphilly and District Workmen's Cottage Hospital. Its patients will be drawn from the ranks of the eleven thousand miners who have established it and will maintain it.

### THE NEW AIR MAILS.

We note from the *Handley-Page Bulletin* that any post office will accept letters, postcards, and papers, whether registered or unregistered, for transmission by air; unregistered packets may also be posted in a public letter-box. The post office charges the ordinary foreign postage, in addition to an air fee of 2d. per ounce for Paris and Brussels, and 3d. per ounce for Amsterdam. If the special blue label supplied free at any post office is not available, the cover may be simply marked "By Air Mail" in the top left-hand corner. For express delivery an extra charge of 6d. is made, and here again the word "Express" must be placed on the packet. As to time-saving, it should be noted that correspondence posted in London in the morning is delivered in Paris or Brussels in the afternoon. Also the air mails save twenty-four hours in the delivery of letters to Switzerland, Italy, Spain, Portugal, Germany, Austria, Sweden, and Denmark.

### WEST BROMWICH HOSPITAL.

The accumulated deficiency on current account of West Bromwich Hospital now amounts to £6,803, and is not far short of the income of the last financial year, which was £8,470. The position, therefore, as stated at the annual meeting, is considered to be rather precarious. The contributors to Hospital Saturday Fund in this locality, as elsewhere, have come to the rescue with very little delay, and have agreed to double their weekly contributions; this example to employers is one which the Board have quickly made use of and they are appealing to those whose workpeople attend the hospital. Another matter which is causing financial worry is the fact that alterations to the nurses' home are hung up for want of means and a grant of £1,000 promised by the Red Cross Society is provisional upon the work being put in hand by the end of August. The Board thought that it would be better to take a certain amount of risk, and the improvements, at an estimated cost of £5,000, have been put in hand, the public being asked to subscribe the remaining £4,000. The medical report shows that the death-rate amongst the patients for the year was 5.1 per cent., and as practically half of these died within forty-eight hours of admission it can be realised how essential a hospital, which can quickly deal with accident cases, is to an industrial centre like West Bromwich; if the facts and figures are brought home to the neighbourhood in a concise and telling way, we are sure that the funds required by the hospital will be quickly forthcoming.

### THE WOOL MERCHANTS OF GOLDEN SQUARE.

In connection with the forthcoming sale of the premises occupied by the Hospital for Diseases of the Throat in Golden Square, it has been pointed out that this locality is now rapidly being largely tenanted by firms interested in the wool trade. Close to Regent Street, Oxford Street, and other important thoroughfares, Golden Square was at one time entirely a residential area, but business houses have gradually elbowed out householders, solicitors and estate agents, in most cases to make room for rolls of woollen goods. In view of the great prosperity of the wool trade and the large prices obtained from the public for everything which has wool as a constituent part, the name "Golden Square" is not inappropriate to what is becoming the chief centre of this particular business, and hospital secretaries may take this hint of a likely field for appeal operations. The Throat Hospital covers an area of 7,000 feet, with three frontages, and the large price which is sure to be obtained for such an important property is likely to be offered by those interested in the trade which has found one of its chief homes in the Square.

### THE ROYAL PRINCE ALFRED HOSPITAL.

That the Prince of Wales, when in Sydney, could not visit the Royal Prince Alfred Hospital was disappointing to the authorities, but some kind of rule had to be made, and the Prince was able to see the purely military hospitals only. A loyal address, however, was presented, and responded to. As there had been some street accidents in Melbourne owing to the large crowds on the occasion of the Royal visit, a ward had been prepared at the Prince Alfred in case history repeated itself at Sydney. Providentially, there was no occasion to make use of the hospital organisation, as, despite the huge crowds, not a single accident occurred. We gather from the imposing volume of 200 pages which is the Prince Alfred Hospital's annual report that money is as difficult to obtain in adequate quantities in Australia as it is at home. If it had not been for the Jubilee Fund, which produced £27,959, the hospital would have been in still lower water. The income for 1920-21 is estimated at £75,000, and the expenditure £95,000; consequently, the directors have been obliged to apply for an increase in the Government subsidy. Payments made by in-patients during the year 1918-19 were £6,766, or, with out-patient fees, £7,557, which is about only ten per cent. of the income.

### FOOTBALL AND CHARITY.

The Arsenal Football Club have sent a donation of twenty-five guineas to the Royal Hospital and Home for Incurables, Putney.

### THE SUPPLY OF OXYGEN.

Because of the difficulty in obtaining speedily a supply of oxygen during the night for serious cases, the Deptford Borough Council is now arranging with the Miller Hospital to supply oxygen at any time when the chemists' shops are shut. At present the experimental arrangement is confined to a small number of cylinders until the demand and the cost have been ascertained.

### AN ELEPHANTINE COLLECTOR.

Theatrical companies have often helped hospitals, but a novel form is the use of an elephant which has been performing at the Grand Theatre, Hanley, to collect money for the North Staffordshire Infirmary and the Crippled Children's Hospital. The animal, which is known as the Carmo elephant, is attached to a touring company, whose generosity deserves to be recorded. There is little doubt that the good offices of the animal will be in request at other centres visited by the company.

### A LESSON FROM CAMBRIDGE TO OXFORD.

The Cambridge and District Workers' Hospital Fund has fulfilled the hope with which it started ten years ago—namely, to raise £1,000 a year for Addenbrooke's and other allied charities. We have watched the fund sympathetically since its foundation. It is one of the earliest examples of an attempt to transplant the penny-a-week contributions of factory centres to the Midland and Southern county towns. It has been successful not only in the shops and businesses of Cambridge but in the neighbouring villages, and sets an example of which the Radcliffe Infirmary, Oxford, and Mr. G. B. Arnshaw would do well to take note, since Oxford is now trying to found a similar fund for the benefit of its hospital. We congratulate Mr. E. J. Papworth, the fund's hon. secretary; and we know one other person in London who will be pleased at this news, Mr. R. J. Coles, the former secretary of Addenbrooke's, who took the keenest interest in its foundation.

### NEW HOSPITAL FOR RYEDALE.

The new Cottage Hospital for Ryedale has been opened by Lady Bell, wife of the Lord-Lieutenant for the West Riding of Yorkshire. The hospital, standing on Helmsley Road, a mile from Kirbymoorside, is built on a site given by Mrs. Reginald Shaw. Mr. Burrows, of Kirbymoorside, is the architect. The hospital, which contains three wards, with two beds each, two bath-rooms, matron's and servants' rooms, is provided through the efforts of Mrs. E. Shaw, of Welburn Manor, Mrs. Holt, and Lady Marjorie Beckett.

### MINERS' HELP FOR WELSH HOSPITAL.

The mining village of Blaina, grouped with its neighbouring townships of Nantyglo and Brynmawr, provides a shining example of what may be accomplished solely by poundage deductions from workmen's wages. Not only was the splendidly-equipped Blaina Hospital erected and is maintained in this manner, but last week saw another striking development of the scheme—namely, the opening of Nevill Hall, Abergavenny, the beautifully situated mansion for many years the Monmouthshire seat of the late Marquess of Abergavenny, as a convalescent home attached to the hospital. The estate purchased comprises some thirty-three acres, and the price, with some redecorations and furnishing, has involved capital expenditure of £22,000, most of which has already been paid-off out of the regular pence contributed by the comparatively small body of workmen. There are nineteen bedrooms at Nevill Hall, and not more than four adults will be put in any one room. There will be ten children's cots, six in one room and four in another. At first provision has been made for fifty patients, but later it will be possible to accommodate sixty or seventy. There is also furnished a very handsome dining-room, a billiard-room, and a room for other games. In the grounds provision has been made for tennis, bowls and croquet. Miss James, from the Blaina Hospital, has been appointed matron of the home.

### HOMESICK SANATORIA PATIENTS.

The Sanatoria Benefit Sub-Committee has reported to the Staffordshire Insurance Committee that fourteen patients, nearly all from Kinver Sanatorium, had taken their own discharge. From the Sub-Committee's inquiries Mr. Kendrick reported the patients in Kinver Sanatorium were advanced cases, most of whom preferred to die at home. It was because of home-sickness that so many patients discharged themselves, and there was no power to detain them if they wished to leave. There was, he said, no complaint against the sanatorium. It was decided that for a period of six months the reasons why patients left should be tabulated for the information of the Committee. The advanced case offers many difficult problems, and the plea that special institutions should be formed for their reception, however desirable on certain grounds, must take this home-sickness into consideration. Meanwhile it is satisfactory to know that no complaint has been made against Kinver Sanatorium.

### A CROWDED MUNICIPAL HOSPITAL.

In view of the excessive number of applications for admission to the municipal hospital for forthcoming confinements, the Willesden Council has been forced to ask the Guardians what accommodation is available at the infirmary. If the Guardians can provide accommodation, the Council will apply to the Ministry of Health to sanction the payment of grants.

### PAYMENT FOR OVERTIME.

Wandsworth Board of Guardians some months ago adopted the principle of a forty-eight-hour week for all the officers in their institutions, with the provision that overtime should be paid to those officers exceeding the forty-eight-hour limit. A long list showed that a large number of officers were entitled to extra pay for overtime work, one officer having put in 188 extra hours and another 200 in three months. At St. James's Infirmary it was found that a much larger number of hours of overtime had been worked than at St. John's Infirmary, but this was accounted for by the fact that at St. James's Infirmary they had suffered enormously from epidemic diseases at a time when the infirmary was enormously full. There is evidently some opposition to the adoption of a forty-eight-hour working week by certain members of the staff, and this may account for the extraordinary number of hours some officers had worked. The principle is good, providing that sufficient officers can be found, for it means that now three are called upon to put in the hours of two before the forty-eight-hour working week was adopted. For many years it has been a scandal the hours Poor-law officers have been compelled to work, and it was full time that it was realised that a nurse could not adequately perform her duties for twelve hours at a stretch. Eight hours on duty is quite sufficient, especially in a Poor-law infirmary, where the type of patient is not always of the best.

### THIS WEEK'S DRUG MARKET.

So far from there being any improvement in business, the depression seems to be more acute; in the main this is due to the unsettled labour outlook, and the market will probably improve when things all round become less unsettled. Stocks in the hands of distributors must in many cases be getting low, but buyers are naturally reluctant to commit themselves at the moment. One of the few articles quoted at a higher figure is strychnine, and in this case the advance does not come as a surprise, in view of the rise in the price of nux vomica. The demand for eucalyptus oil is still in evidence, and the price tendency is still in an upward direction. Among the articles which have moved downwards in price are benzoic acid, sodium benzoate, barbitone, citric acid, castor oil, chloral hydrate, Japanese refined camphor, resorcin, and phenazone. The accumulation of stocks of potassium bromide have resulted in offers on a lower basis.

